# Update on the Development of SUVN‐I6107: A True Positive Allosteric Modulator of the Muscarinic M1 Acetylcholine Receptor for the Treatment of Dementia

**DOI:** 10.1002/alz70856_099660

**Published:** 2025-12-25

**Authors:** Vijay Benade, Anil Shinde, Rajesh Kumar Badange, Mohammed Abdul Rasheed, Ramkumar Subramanian, Surendra Petlu, Renny Abraham, Vinod Kumar Goyal, Veera Raghava Chowdary Palacharla, Venkatesh Goura, Santosh Kumar Pandey, Rajesh Babu Medapati, Rajesh Kallepalli, Ramakrishna Nirogi

**Affiliations:** ^1^ Suven Life Sciences Ltd, Hyderabad, Telangana, India; ^2^ Suven Life Sciences, Hyderabad, Telangana, India

## Abstract

**Background:**

SUVN‐I6107 is a novel and selective muscarinic M1 Positive Allosteric Modulator (PAM) being developed for the treatment of dementia due to neurodegenerative disorders. In the current research, the pharmacological properties of SUVN‐I6107 in various animal models of cognitive deficits were investigated.

**Methods:**

SUVN‐I6107 was characterized using a calcium mobilization assay and the binding affinity towards the orthosteric M1 ‐ M5 site was investigated to assess the selectivity. The effect of SUVN‐I6107 on neuronal spike rate in coronal hippocampal slice electrophysiology was studied in agonist and PAM modes of testing. The pharmacokinetic properties of SUVN‐I6107 were studied both in rodent and non‐rodent species. The effect of SUVN‐I6107 on MK‐801 (antagonist of the NMDA receptor) induced memory deficits in rats using object recognition task (ORT) and delay‐induced social memory deficits in rats using social recognition task (SRT) was studied. Furthermore, effects of SUVN‐I6107 on neuronal markers like soluble amyloid precursor protein (sAPPα) and inositol 1 phosphate (IP‐1) levels were studied in rats.

**Results:**

SUVN‐I6107 showed allosteric potency at M1 receptor (EC_50_ of 355 nM) with improved neuronal firing, when tested in combination with EC_20_ of carbachol. SUVN‐I6107 showed good oral bioavailability in rats, dogs, and monkeys. SUVN‐I6107 was found to have brain penetration properties with adequate protein‐free fraction. In a rat model, SUVN‐I6107 has reversed the delay, scopolamine and MK‐801 induced amnesias in ORT. SUVN‐I6107 also significantly improved the memory in contextual fear conditioning task and SRT. Treatment with SUVN‐I6107 produced significant increase in levels of cortical sAPPα and striatal IP‐1 in rats.

**Conclusions:**

Results from the non‐clinical studies suggest SUVN‐I6107 may have memory enhancing property in various forms of dementias. SUVN‐I6107 is currently being studied in a Phase‐1 study (NCT06705088) to evaluate its safety, tolerability, pharmacokinetics, and pharmacodynamic effects after single and repeated administrations in healthy human subjects.

